# The Berg Balance Scale at Admission Can Predict Community Ambulation at Discharge in Patients with Stroke

**DOI:** 10.3390/medicina57060556

**Published:** 2021-05-31

**Authors:** Wen-Ling Liao, Chiung-Wen Chang, Pi-Yu Sung, Wei-Nung Hsu, Ming-Wei Lai, Sen-Wei Tsai

**Affiliations:** 1 Department of Physical Medicine and Rehabilitation, Taichung Tzu Chi Hospital, Buddhist Tzu Chi Medical Foundation, Taichung City 427, Taiwan; wling00921@gmail.com (W.-L.L.); m645490@yahoo.com.tw (C.-W.C.); sungpiyu@gmail.com (P.-Y.S.); Hsuweinung@gmail.com (W.-N.H.); mweivv@gmail.com (M.-W.L.); 2 Department of Post-Acute Care Center, Taichung Tzu Chi Hospital, Buddhist Tzu Chi Medical Foundation, Taichung City 427, Taiwan; 3 School of Medicine, Tzu Chi University, Hualien 970, Taiwan

**Keywords:** Berg Balance Scale, ambulation, stroke, post-acute care

## Abstract

*Background and Objectives*: To regain the ability of community ambulation is a meaningful goal for stroke patients. Recent research recommended that the distance accomplished during the six-minute walk test (≥205 m in 6MWT) is the fittest for defining community ambulation. Until now, there are few studies that have used the updated definition to investigate the related predictors. The aim of this study was to investigate the association between the admission clinical parameters and community ambulation measured by the 6MWT at discharge. The other aim was to find the admission Berg Balance Scale (BBS) cut-off score to discriminate between household or community ambulators. *Materials and Methods*: This cohort study collected the data of patients who entered the post-acute Care Cerebrovascular Diseases program. Multivariate logistic regression was used to identify significant predictors measured at admission that are associated with community ambulation, and a receiver operating characteristic was adopted to calculate the cut-off value for admission status. There were 120 participants included in this study, and 25% (*n* = 30) of them regained the ability of community ambulation at discharge. The BBS on admission was identified as the only significant predictor for community ambulation (odds ratio 1.06). *Results*: The optimal cut-off score for the BBS at admission was 29, and the area under the curve for BBS scores on admission when discriminating between household and community ambulators at discharge was 0.74. *Conclusions*: The admission BBS scores could be used to predict household and community ambulators at discharge in stroke patients. The results of this study could help clinical physicians set appropriate discharge goals early.

## 1. Introduction

Achieving independent ambulation ability is a primary goal of patients post-stroke [1,2]. Walking capacity has been found to be reduced in patients following a stroke, and the loss of independent ambulation is perceived as the most disabling consequence of a stroke [3]. In particular, post-stroke patients who cannot achieve community ambulation are more dependent on family and friends [4] and have lower scores for health-related quality of life [5].

Previous studies have found several factors associated with walking ability in stroke patients including age, time post-stroke, cognitive function, balance, and walking speed. However, balance is the most frequently mentioned predictor of community ambulation in stroke patients [6,7,8]. The Berg Balance Scale (BBS) is one of the most widely used tools for balance assessment in the rehabilitation settings. For stroke patients who are unable to walk in the preliminary stage, the BBS could be used to measure their balance status. Previous studies have shown the association between the BBS score and the ability of community ambulation, which was defined by walking speed, in stroke patients [9,10]. However, Fulk et al. [11] recommend that the distance of ≥205 m accomplished in the 6MWT is more suitable than walking speed for defining community ambulation ability. Until now, there has been a lack of studies that have used the updated definition to investigate whether or not the BBS can still be used as a predictor in post-acute rehabilitation settings for community ambulation at discharge.

In Taiwan since 2014, post-acute stroke care has been standardized by Taiwan National Health Insurance (TNHI) through the Post-acute Care Cerebrovascular Diseases (PAC-CVD) program. The aim of the PAC-CVD program is to accelerate the functional recovery in stroke patients by providing comprehensive rehabilitation plans, including multidisciplinary interventions and advice about discharge plans [12] in order to reduce the burden of caregivers, the length of stay and to reduce the medical costs. After receiving the PAC-CVD program, patients are encouraged to be discharged home. Therefore, it is important to determine whether patients could regain the community walking ability at discharge upon being enrolled in the program.

Based on the patients admitted to the PAC-CVD program in a single center, the aim of the present study was to investigate the association between the clinical predictors measured in admission and community ambulation as defined by the distance accomplished during the 6MWT at discharge. The second aim was to explore the BBS cut-off score at admission to discriminate between household and community ambulators.

## 2. Materials and Methods

### 2.1. Participants and Settings

This retrospective cohort study collected the data of patients who entered the PAC-CVD program implemented by TNHI in Taichung Tzu Chi Hospital, Taiwan. From October 2014 to November 2019, all consecutive patients transferred to the Taichung Tzu Chi PAC unit who met the following inclusion criteria were recruited: (1) patients who had suffered from an acute stroke within one month; (2) patients under stable medical conditions and with the functional level of an mRS score of 2–4; (3) patients with the potential to receive intensive rehabilitation programs who could actively participate in the treatment. In order to be able to follow up the walking recovery pattern in stroke patients, we listed the length of stay as less than 6 weeks in the PAC unit as one of the exclusion criteria. The other exclusion criteria were as follows: (1) 6MWT at admission ≥205 m; (2) discontinued the program; (3) severe complications such as recurrent stroke, myocardial infarction, or other medical situation that prevented the patient from receiving an intensive rehabilitation program; (4) loss of complete assessment data.

Based on the TNHI regulations, patients who are enrolled in the PAC-CVD program should receive comprehensive assessments including various aspects of body function and activity every three weeks from admission to discharge. The comprehensive assessments were administered by a care team composed of physicians, physical therapists, occupational therapists, speech therapists, nutritionists, pharmacists, nurses, case managers, and social workers, and all of them received training courses about the assessment tools. This study was approved by the ethics committee of Taichung Tzu Chi Hospital.

### 2.2. Rehabilitation

All patients in the PAC-CVD program received a highly intensive rehabilitation program of physical therapy, occupational therapy, or speech therapy for two to three hours based on the patients’ status, 6 days per week. The contents of this highly intensive PAC-CVD rehabilitation were dependent on the patient’s ability, including 2–3 h of an intensive rehabilitation program each treatment day, and the treatment goals were established by the patient and our multidisciplinary rehabilitation team. The maximum duration of the PAC-CVD hospital stay was limited to 12 weeks.

### 2.3. Primary Outcomes

The primary outcome of this study was whether the participants had the ability to ambulate in the community at discharge. According to a study by Fulk et al. [11], patients who achieved ≥205 m during the 6MWT were classified as community ambulators; participants who did not reach this distance were regarded as household ambulators. The reliability and validity of the 6MWT used in stroke inpatient rehabilitation has been validated in previous studies [13,14]. During the 6MWT, patients were asked to walk along a 30 m walkway and turnaround the markers as many times as they could in six minutes [15].

### 2.4. Independent Variables

Several factors were collected immediately after admission to investigate the ability to predict walking ability at discharge. The scales used in this study included the BBS, the 5-Meter Walk Test (5MWT), the Mini Mental State Examination (MMSE), and the Mini Nutritional Assessment (MNA). These scales are commonly used in clinical settings to measure the performances of stroke patients in various aspects and to monitor the recovery status of the patients. Previous studies have confirmed the reliability and validity of these scales for stroke patients in the post-acute stage [14,16,17].

The BBS was used to measure functional balance ability; this is a 14-item scale with tasks to assess static and dynamic balance in sitting and standing [18]. The BBS scores are related to length of stay, discharge destination, and disability level in stroke patients [16]. The BBS score ranges from 0 to 56, and a higher score means a better balance ability.

The 5-Meter Walk Test (5MWT) was adopted to evaluate the participants’ fast walking speed [19]. During the 5MWT, the participant should walk without any physical assistance; however, supervision from the assessors was allowed. If the patient could not walk without physical assistance, then the speed of the 5MWT was recorded as zero. The Mini Mental State Examination (MMSE) was used to screen for cognitive impairment. The score of the MMSE ranges from 0 to 30, and a higher score means a better cognitive status [20]. The Mini Nutritional Assessment (MNA) was adopted to evaluate the nutritional status of participants. The score of the MNA reflects whether participants are at risk from malnutrition and is related to mortality and hospital cost in the elderly [21].

Demographic characteristics such as age, gender, type of stroke (cerebral infarction or cerebral hemorrhage), nasogastric tube or urinary catheter used at admission, days between stroke onset and admission, and length of stay in the PAC-CVD program were included in the analysis.

### 2.5. Statistical Analysis

Participants were classified as household or community ambulators based on the distance accomplished in the 6MWT at discharge. Mean and standard deviation (SD) were reported for the variables assessed at admission.

A univariate analysis was conducted to compare the variables at admission between the groups of home and community ambulators. The Mann–Whitney U test was used for continuous variables and a χ^2^ test or Fisher’s exact test was conducted for the categorical variables to determine whether any significant differences existed between the two groups.

Significant factors in the univariate analysis were included in a multivariate logistic regression analysis to determine the significant independent variables at admission for predicting community ambulation at discharge. Receiver operating characteristic (ROC) curves were used to plot the figure of sensitivity against 1- specificity. The area under the ROC curve (AUC) was calculated to determine whether the admission BBS had the predictive ability to identify household or community ambulators at discharge, and then Youden’s index was used to identify the optimal cut-off value for the BBS at admission. We conducted 2-sided statistical analyses for all of the above analyses and set *p* < 0.05 as the statistically significant level.

## 3. Results

**Figure 1:**
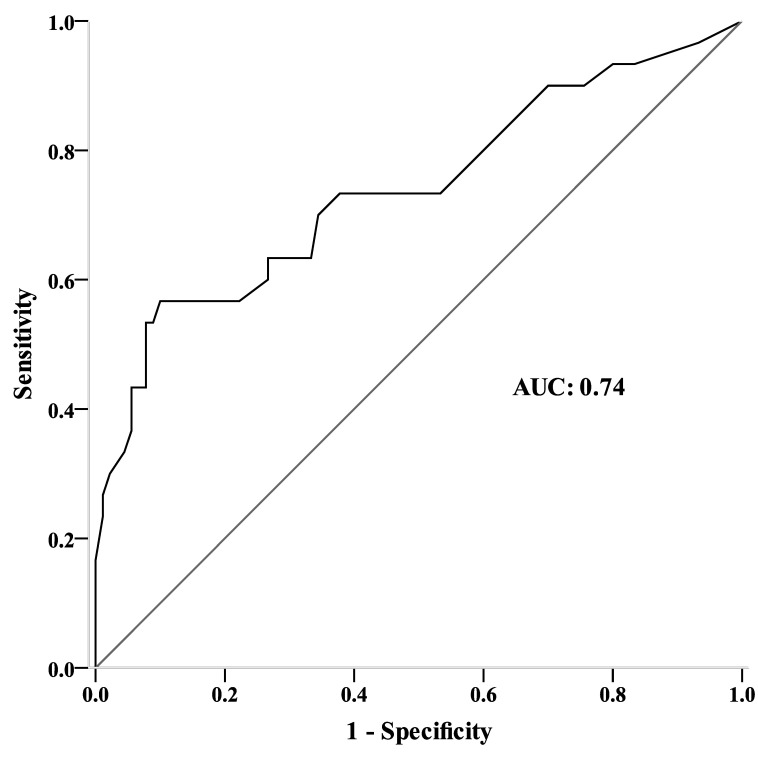
Receiver operating characteristic curve analysis of the Berg Balance Scale (BBS) score at admission for predicting community ambulation ability at discharge (6MWT distance ≥ 205 m).

During the data collection period, 312 stroke patients were admitted to our PAC unit and 192 were excluded based on the exclusion criteria. One hundred and twenty participants were included in this study, and no one dropped out from the study. At discharge, 25% (*n* = 30) of them regained the ability of community ambulation (Figure 1). Table 1 lists the characteristics at admission of total and partitioned participants based on household (*n* = 90) or community (*n* = 30) ambulators at discharge. The mean age of all participants was 66.46 years, and the mean length of stay in the PAC-CVD program was 67.94 days. Patients in the group of community ambulators were significantly younger than those in the group of household ambulators (*p* = 0.05). According to the admission assessment, the group of community ambulators had significantly higher scores on the BBS and the 5MWT.

Table 2 shows the results of the multivariate logistic regression analysis. The significant factors found in the univariate analysis included age, the BBS, and the 5MWT and were used in the multivariate logistic regression analysis. In the multivariate analysis, admission BBS was identified as the only significant predictor for community ambulation (*p* = 0.00), and the odds ratio (OR) was 1.06 (95% confidence interval [CI], 1.02–1.10).

Figure 1 plots the ROC curves of the BBS scores at admission in predicting community ambulation ability at discharge. Table 3 shows that the optimal cut-off score for the BBS at admission was 29 (sensitivity 57%, specificity 90%, positive likelihood ratio [LR+] 5.67, and [LR−] 0.48), and the area under the ROC curves for the admission BBS scores in discriminating between household and community ambulators at discharge was 0.74 (95% CI, 0.62–0.86).

## 4. Discussion

This study found that younger age, better BBS scores, and a faster 5MWT gait speed at admission were factors associated with community ambulation at discharge. However, after the multivariate logistic regression analysis, the BBS score at admission was identified as the only significant predictor for community ambulation. Furthermore, the result of the ROC curve analysis showed that the cut-off score of 29 for the BBS at admission could be used to predict the community ambulation ability at discharge in stroke patients. 

The BBS is a widely used scale in clinical settings and is listed as the primary tool to monitor the balance progression of stroke patients in the TNHI PAC-CVD program. The established BBS cut-off score at admission used to predict discharge community ambulation in our study has several clinical implications. For example, clinicians may choose to increase the dosage of balance and gait training including walking on stairs, slopes or uneven surfaces for those who scored 29 or higher on the BBS at admission, whereas clinicians may decide to emphasize the skills needed to use a wheelchair outdoors for the patients with BBS scores of less than 29 at admission. Based on the study findings, the rehabilitation team could set the appropriate intervention plans and discharge goals early according to the results of admission measures [9].

This study showed that participants who regained the ability of community ambulation at discharge were younger, and it suggested that younger post-stroke patients may regain better mobility outcomes in the TNHI PAC-CVD program. This is consistent with the research of Durcan and colleagues that showed that age was significantly associated with community ambulation [22]. Furthermore, Bindawas et al. concluded that older adults had limited functional outcomes after a stroke [23]. As age increases, physical fitness such as muscle strength, cardiopulmonary fitness, and flexibility tend to be reduced [24,25]. The age-related differences in physical activity may explain why the age in the community group was younger than in the home ambulation group. 

In our study, although the gait speed was significantly faster on admission in the group of community ambulators when compared with home ambulators, gait speed was not a significant predictor associated with community ambulation in the multivariate logistic regression analysis. The results of the study were similar to the previous study which found that standing balance significantly confounded the relationship between gait speed and community ambulation [8]. The above information may mean that balance ability is a stronger factor than gait speed in predicting community ambulation.

Although previous studies have investigated the relationship between the BBS score and the ability of community ambulation in stroke patients, the definitions of community ambulation are different among these studies. Louie et al. [9] defined gait speed at or faster than 0.80 m/s as regaining community ambulation, whereas Bland et al. [10] adopted a lower speed (0.40 m/s) to discriminate between household and community ambulation. Based on the different definitions of community ambulation, various predictive cut-off scores on the BBS on admission have been reported. Louie et al. [9] identified a cut-off score of 29 on the BBS, which was suggested as a predictor for community ambulation following rehabilitation, while Bland et al. regarded the combination of a BBS score of 20 on admission and a Functional Independence Measure walk item score of 1 or 2 as the discriminative criteria. Although gait speed is typically used to categorize different functional walking abilities, Fulk et al. [11] identified the distance of ≥205 m accomplished in the 6MWT as the strongest individual factor related to community ambulation ability in stroke patients and suggested that the functional walking categorization defined by gait speed may overestimate actual walking abilities. Based on the above reasons, we adopted the 6MWT as the outcome measure and found that a cut-off BBS score of 29 on admission was predictive of regaining community ambulation.

The BBS cut-off scores present in this study are the same as those in the study by Louie et al. but [9], there are several different points between both studies. First, the definitions of community walking are different as mentioned above. Second, the follow-up time is distinct. The end of the tracking time in the study by Louie et al. is 6–7 weeks post-stroke whereas in this study the end of the tracking time is 11–12 weeks post-stroke. According to previous research [26], 6–7 weeks post-stroke is not enough time to observe the maximum walking recovery in stroke patients. Furthermore, the participants in the study by Louie et al. were included from another randomized controlled trial which may have confounded the study results.

There are some limitations in this study. First, the generalization of the study findings meant that we limited our study to those who met the inclusion and exclusion criteria listed in our study, such as suffering from a first-ever acute stroke, an mRS score of 2–4, and a stable medical condition. Second, the study design was a retrospective analysis, which is a drawback. Third, some variables that may be related to community mobility in stroke patients were not included in our study, such as motor function of the lower extremities, fear of falling, and the presence of depressive symptoms. Furthermore, in addition to walking endurance, community ambulation ability may be affected by other aspects such as motor function or psychosocial status, which were not included in our study.

This is the first study using the distance accomplished in the 6MWT to define community ambulation and find the BBS cut-off score. A previous study showed that maximum walking recovery will be accomplished in the first 11 weeks post-stroke [26], and our study investigated the fastest recovery period that would be helpful for clinical physicians to plan different treatment programs and set appropriate discharge goals based on the assessments at rehabilitation admission. However, to identify whether a patient could regain the community ambulation ability at discharge is a complex issue involving physical, environmental, and personal factors. In addition to the walking distance, clinicians should take into account other influencing factors such as environmental barriers, self-efficacy, or the subjective perspective of participation in community walking to provide a comprehensive care plan [27].

In conclusions, our findings suggest that if the patients score 29 or higher on the BBS at admission to the PAC-CVD program, then they are highly likely to regain the ability to walk in the community by 11 weeks post-stroke.

## Figures and Tables

**Table 1:** Participant characteristics at admission.

Variable	Total (*n* = 120)	Household Ambulator at Discharge (*n* = 90)	Community Ambulator at Discharge (*n* = 30)	*p*-Value
Age (years)	66.46 ± 13.61	67.81 ± 13.86	62.40 ± 12.15	0.05
Gender (female/male) (%)	56 (47)/64(53)	44 (49)/46(51)	12 (40)/18(60)	0.40
Type of stroke (cerebral infarction/cerebral hemorrhage) (%)	91 (76)/29 (24)	69 (77)/21(23)	21 (70)/8(30)	0.71
Days between stroke onset and admission	14.67 ± 6.81	14.59 ± 7.29	14.90 ± 5.20	0.83
Length of stay (days)	67.94 ± 13.72	68.07 ± 13.58	67.57 ± 14.34	0.86
Nasogastric tube used at admission (yes/no) (%)	25 (21)/95 (79)	20 (22)/70 (78)	5 (17)/25 (83)	0.52
Foley catheter used at admission (yes/no) (%)	9 (8)/111 (92)	9 (10)/81 (90)	0 (0)/31 (100)	0.11
BBS	15.38 ± 14.03	11.98 ± 11.25	25.57 ± 16.62	<0.00 *
5MWT (m/s)	0.10 ± 0.20	0.06 ± 0.15	0.22 ± 0.27	0.01 *
MMSE	20.38 ± 8.56	19.84 ± 8.79	21.97 ± 7.76	0.24
MNA	11.89 ± 5.67	12.01 ± 5.91	11.53 ± 4.96	0.67

* *p* < 0.05. Values expressed as the mean ± SD or n. BBS: Berg Balance Scale; 5MWT: 5-Meter Walk Test; MMSE: Mini-Mental State Examination; MNA: Mini Nutritional Assessment; SD: standard deviation.

**Table 2:** Multivariate logistic regression model to predict community ambulation at discharge.

Variable	B	OR (95% CI)	*p*-Value
Age	−0.03	0.97 (0.94–1.01)	0.14
BBS	0.06	1.06 (1.02–1.10)	0.00
5MWT	1.21	3.36 (0.30–38.41)	0.33

BBS: Berg Balance Scale; 5MWT: 5-Meter Walk Test; B: regression coefficient; OR: odds ratio; CI: confidence interval.

**Table 3:** The optimal Berg Balance Scale cut-off scores at admission for community ambulation at discharge determined from the ROC curve.

BBS Cut-Off Score	Sensitivity	Specificity	Positive Likelihood Ratio	Negative Likelihood Ratio	Area under the ROC Curves (95% CI)
≥29	0.57	0.90	5.67	0.48	0.74 (0.62–0.86)

ROC: receiver operating characteristic; BBS: Berg Balance Scale; CI: confidence interval.

## Data Availability

The data presented in this study are available on request from the corresponding author. The data are not publicly available due to data were collected from patients who entered the PAC-CVD program in Taichung Tzu Chi Hospital.
